# Plant microRNA-Target Interaction Identification Model Based on the Integration of Prediction Tools and Support Vector Machine

**DOI:** 10.1371/journal.pone.0103181

**Published:** 2014-07-22

**Authors:** Jun Meng, Lin Shi, Yushi Luan

**Affiliations:** 1 School of Computer Science and Technology, Dalian University of Technology, Dalian, Liaoning, China; 2 School of Life Science and Biotechnology, Dalian University of Technology, Dalian, Liaoning, China; International Centre for Genetic Engineering and Biotechnology (ICGEB), India

## Abstract

**Background:**

Confident identification of microRNA-target interactions is significant for studying the function of microRNA (miRNA). Although some computational miRNA target prediction methods have been proposed for plants, results of various methods tend to be inconsistent and usually lead to more false positive. To address these issues, we developed an integrated model for identifying plant miRNA–target interactions.

**Results:**

Three online miRNA target prediction toolkits and machine learning algorithms were integrated to identify and analyze *Arabidopsis thaliana* miRNA-target interactions. Principle component analysis (PCA) feature extraction and self-training technology were introduced to improve the performance. Results showed that the proposed model outperformed the previously existing methods. The results were validated by using degradome sequencing supported *Arabidopsis thaliana* miRNA-target interactions. The proposed model constructed on *Arabidopsis thaliana* was run over *Oryza sativa* and *Vitis vinifera* to demonstrate that our model is effective for other plant species.

**Conclusions:**

The integrated model of online predictors and local PCA-SVM classifier gained credible and high quality miRNA-target interactions. The supervised learning algorithm of PCA-SVM classifier was employed in plant miRNA target identification for the first time. Its performance can be substantially improved if more experimentally proved training samples are provided.

## Introduction

MicroRNAs (miRNAs) are a large family of small endogenous noncoding RNAs with a length of 20–24 nucleotides (nt). They have significant regulatory functions in plants and animals [1]. Unlike other small RNAs, miRNAs undergo a distinctive biogenesis containing a transcript folding back step to constitute a characteristic stem-loop structure [2]. Pre-miRNAs are processed from the stem-loop transcripts mainly by RNase III endonucleases enzyme Drosha or Dicer-like 1 (DCL1) [3], [4]. Then, another Dicer or DCL1 enzyme participates in cutting pre-miRNAs into miRNA:miRNA* double strands. Finally, helicase enzymes in cytoplasm separate the double strand into two single strands. One of them combines with an Argonaute protein and forms the RNA-induced silencing complex (RISC) [5]. Since the first miRNA was discovered in *C. elegans* at the end of last century [6], thousands of miRNAs have been identified by using computational and molecular approaches.

The regulation of miRNAs is exerted by complementary base-pairing to the target mRNA, based on which the identification of miRNA-target interactions has been widely performed. It is most likely that miRNA targets play an indispensable role in many aspects involved in the development or response to the environment [7]. By studying the location and certain time of the regulation of a target from miRNA, we can further understand both the regulation of gene and system biology. Usually, miRNAs regulate posttranslational repression of mRNAs via two different mechanisms. Firstly, the miRNAs induce mRNA translational repression, sometimes coupled with accelerated mRNA decay, by the inhibition of the translation initiation or poly(A) shortening [1], [8]. Secondly, with high complementarity between miRNAs and targets, the miRNAs induce mRNA cleavage under the help of Argonaute protein [1], [9]. Unlike animals, the complementarity between plant miRNA and target tends to be near-perfect and therefore improves the effectiveness and reliability of computational predictions [10].

Currently, a large amount of plant miRNAs have been discovered and reported with the development of high throughput screening techniques. Besides, the machine leaning technique also makes great contribution to the prediction of probable mature miRNAs [11], [12]. Meanwhile, lots of efforts have been made to identify miRNA-target interactions. For example, a latest study has successfully identified 119 targets in *Solanum lycopersicum*, 106 of which appeared to be new [13]. However, although a certain amount of miRNA targets have been identified and experimentally validated, this issue is far from settled. Firstly, more efficient and reliable prediction tools are required to solve the challenge caused by the rapidly increasing scale of miRNAs. Secondly, the reported miRNA targets are far less than the existing. Besides, a mass of miRNA targets still remain to be deliberated.

Computational prediction approaches have made a great contribution to identify miRNA-target interactions [14]. Here we divide the existing target prediction methods into two categories: statistical prediction and machine learning approaches. Features used in the first category can be summarized as followings: (i) binding site evolutionary conservation, (ii) complementarity between miRNA and target site, and (iii) target site accessibility. Methods based on these features are widely applied in both animals and plants. Outcomes of these predictors are credible to some extent with acceptable computational complexity. Representative programs of this category for plants are miRU [15], psRNATarget [16], UEA toolkit [17], TargetFinder [18], TAPIR [19], et al. Although these predictors have been widely employed, it is still unclear to date how these factors could influence the recognition mechanism. Programs belonging to the first category, considering parts or all of the three features, lack comprehensive consideration which may lead to more false positive or negative predictions [14]. Furthermore, targets may be missed due to the undue dependence on conservation information. To integrate these multiple factors and reduce false positive rate effectively, the second category of prediction methods introduce machine learning algorithms. Unlike statistical prediction approaches, algorithms of this category use known miRNA-target interactions and incorporate the degradome and transcriptome data in an efficient way. Usually, a classifier model is trained using these known miRNA-target interactions to predict suspected ones. Machine learning approaches have already been employed in animal miRNA targets prediction successfully, such as miTarget [20], GenMiR++ [21], mirTar [22] and RNA22 [23]. P-TAREF [24] is a successful tool implementing Support Vector Regression (SVR) approach for the identification of plant miRNA targets. However, machine learning techniques have not been used in this field maturely.

An integrated model is presented to identify the miRNA-target interactions of *Arabidopsis thaliana*, the most studied plant species, using both categories of methods aforementioned. It contains three credible online predictors which provide preliminary miRNA targets as candidates. In order to reduce the false positive candidates, a self-training based PCA-SVM classifier is applied using a priori knowledge. The integrated model takes advantage of the two categories of technologies and thus produces higher quality of miRNA-target interactions. Meanwhile, degradome data is employed to confirm the reliability of our predicting outcomes. Results show that our integrated approach gains more credible miRNA-target interactions. Furthermore, the proposed approach is performed over *Oryza sativa* and *Vitis vinifera* to prove the applicability of our approach. The tool of our research is available in our supporting website: http://pan.baidu.com/s/1pJLR1nt.

## Methods

**Figure 1:**
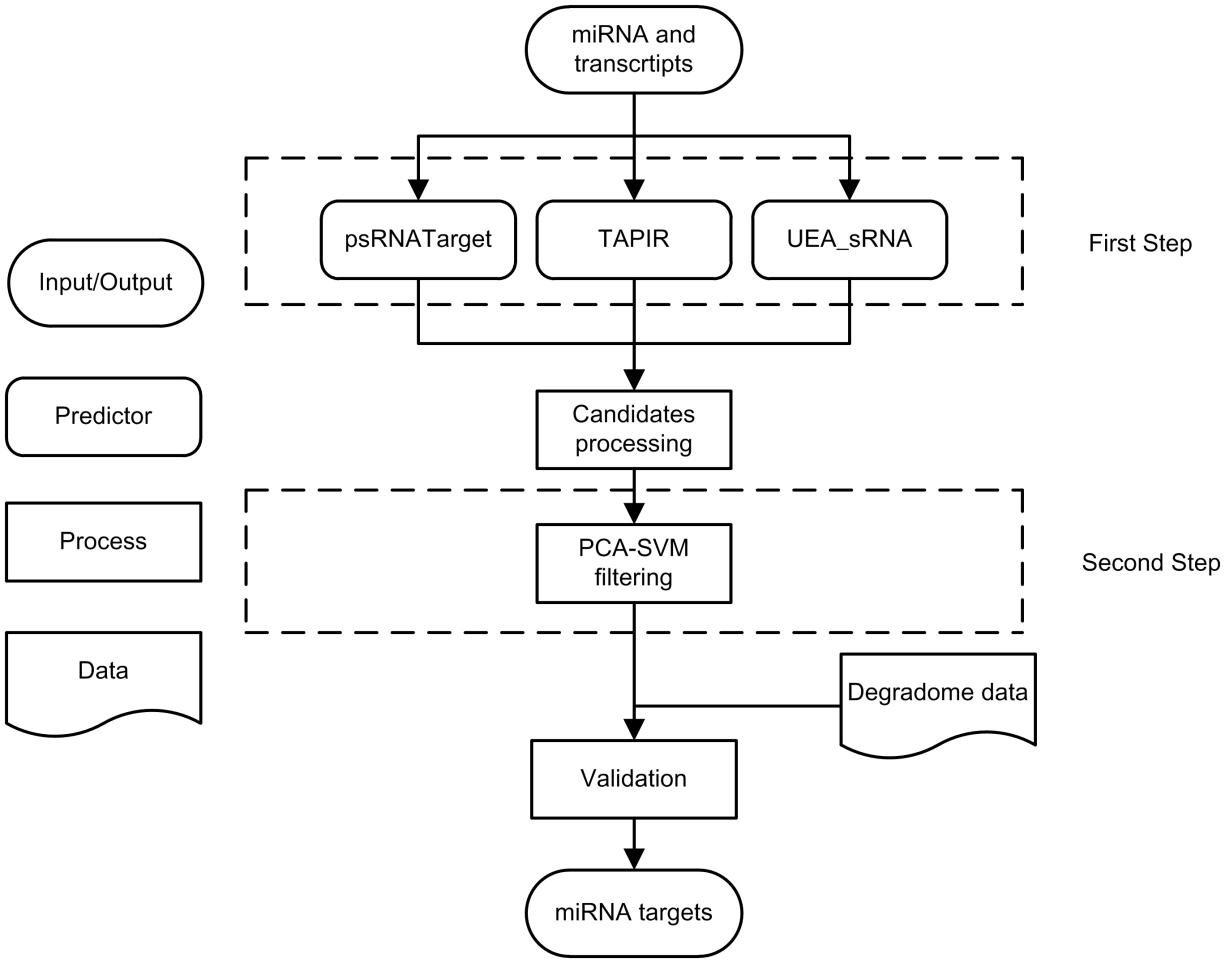
The pipeline of the whole approach. Our approach is mainly divided into two steps: Online prediction and local classification. PsRNATarget, TAPIR, UEA_sRNA were chosen to predict original miRNA target candidates in the first step. 99 experimentally validated miRNA target interactions are employed to serve as SVM positives in the second step. Moreover, 1618 degradome sequencing supported miRNA target interactions are collected for validation experiment.

Three widely used prediction methods were integrated with a SVM-based local classifier, aiming to obtain high quality miRNA targets. The whole process is shown in Figure 1. The first step is to predict target candidates using online predictors. miRNAs and transcript sequences are uploaded to online predictors respectively for target searching. Results are locally stored and processed into unified format as a primary candidate set. Secondly, semi-supervised learning algorithm, PCA-SVM, is adopted to classify the primary candidate set to separate more credible candidates from the false positives. During the training of SVM model, experimentally validated miRNA-target interactions are applied to act as positives. A same number of negatives are randomly picked from the result sets supported by the three single predictors, which represent less credible ones gained by only one predictor. This part contributes to the reducing of false positive rate and ensures the reliability of miRNA-target interactions gained by our approach. Besides, the used SVM outdoes other classification model, e.g. Naive Bayesian Model and Random Forest Model. Finally, a validation experiment with degradome-seq data is implemented to confirm the reliability of the output miRNA-target interactions information.

### Dataset

The transcript sequences (5′UTR, CDS and 3′UTR) of *Arabidopsis thaliana* were downloaded from the central database TAIR [25] (http://www.arabidopsis.org/, Release 9). And 338 *Arabidopsis thaliana* mature miRNAs arising from 299 pre-miRNAs were obtained from the miRNA database miRBase [26] (http://www.mirbase.org/, Release 19). The *Oryza* RNA sequences came from Ensembl Genomes (http://plants.ensembl.org/, Release 15) and RNA sequences for *Vitis vinifera* were downloaded from FTP site of JGI genomic project (http://genome.jgi-psf.org/, version 9). All miRNAs for *Oryza sativa* and *Vitis vinifera* also came from miRBase.

The *Arabidopsis thaliana* training data set of positives and negatives in our SVM model were gained by different ways. The positives comprise experimentally validated miRNA-target interactions, which have precise identification information of the binding sites. A total of 99 positives reported in previous studies [27]–[30] were collected. We defined the candidates predicted by only one online predictor only. This means that these targets are not supported by other predictors, as the negatives. The reasons and methods will be introduced in detail later.

Degradome-seq [27] and CLIP-Seq [31] are two effective methods employed for miRNA-target identifications. To evaluate the performance of our approach, we downloaded 1618 *Arabidopsis thaliana* miRNA-target interactions data supported by degradome sequencing from starBase [32] (http://starbase.sysu.edu.cn/, Release 2.1). The results are generated via CleaveLand (version 2.0) software [33] with a default Penalty Score of 4.5. These sequences are only used in the validation step for lacking of precise binding information.

### Online predictors

We analyzed the results of widely used predictors profoundly and found that there are usually inconsistences between different results sets. This is mainly because of various rules and strategies used in the predictors. In order to gain a more comprehensive result set, the combination of different predictors were used. psRNATarget, TAPIR and UEA were chosen to predict miRNA target candidates in the first step showed in Table 1. They are widely used statistical prediction tools in integrated prediction approaches [34], [35] which relying on different combinations of seed pairing, central pairing, and hybridization energy of target site. Detailed rules and strategies used in these predictors are inconsistent to some degree.

**Table 1:** Information of three online predictors for plant miRNA targets.

Method	link	AUTS^a^	Limit^b^	Spe^c^
**psRNATarget**	http://plantgrn.noble.org/psRNATarget/?function=3	Y	20M/200M	< = 5 min
**TAPIR**	http://bioinformatics.psb.ugent.be/webtools/tapir/	Y	50 kb/40M	5–30 min
**UEA_sRNA**	http://srna-tools.cmp.uea.ac.uk/plant/cgi-bin/srna-tools.cgi?rm=input_form&tool=target	N	50miRs/None	> = 1 hour

a Accepttion of user-supplied transcripts.

b Limitation for miRNA/transcript input.

c Approximate running time.

psRNATarget [16] is a plant sRNA (miRNA/siRNA) target analysis server, which features two analysis functions: reverse complementary matching and target-site accessibility evaluation. The scoring scheme used in this tool is originally applied by miRU [15]. Instead of using the NCBI BLAST program, psRNATarget employed SSEARCH (Version 36.x), a Smith-Waterman [36] based implementation. Moreover, the server runs on a Linux cluster with an efficient distributed computing back-end pipeline. Therefore, it can be used to analyze high-throughput and next-generation data rapidly.

TAPIR [19] offers the possibility to search for plant miRNA targets using a fast (FASTA) search engine and a precise (RNAhybrid) search engine. Users can choose the precise option to guarantee more imperfectly paired miRNA target duplexes, gained with a much slower speed. The score calculated for each miRNA target duplex came from previous studies [28]. Mismatches, gaps, bulges and GU wobbles are considered here and the weights of them vary inside and outside the core region. Considering the speed, we prefer the fast FASTA search engine.

UEA_sRNA is a method included in the UEA toolkit [17] aiming to identify sRNA targeted transcripts. According to previous studies [28], [37], it focuses on mismatches belonging to different areas of the miRNA target duplex including GU wobbles and adjacent mismatches. MFE (minimum free energy) was computed as an evaluation criterion instead of traditional optimal energy. Comparing with Targetfinder, which uses similar rules, we give preference to UEA_sRNA to search miRNA-target interactions on genome-wide.

The proposed approach used the combination of three predictors. For psRNATarget and TAPIR, 338 *Arabidopsis thaliana* mature miRNAs and transcipts in TAIR9 were uploaded. In the prediction of UEA_sRNA, miRNAs and selected TAIR9 dataset is provided. To keep the balance between the number of candidates and false positive percentage, these predictions were executed via default score cutoff. Detailed values of the parameters are given in Figure S1. The primary miRNA target candidate set was composed of the results from all three preceptors. In the candidates processing module, the result of UEA_sRNA was double checked. Some detailed information was corrected because the transcripts offered by UEA_sRNA had some slight inconsistency with what we used. Moreover, redundant information was removed and elements of the candidate set were simply marked with their origin and stored locally for further use and analysis.

To facilitate the analysis, we defined and considered the following subsets:

1. Outside Subset (OS): Containing parts of candidate set supported by single predictor.
2. Middle Subset (MS): Containing parts of candidate set supported by only two predictors.
3. Inside Subset (IS): Containing parts of candidate set supported by all three predictors.
4. Whole Subset (WS): The whole candidate set supported consisting of the union of OS, MS and IS.

### SVM classifier

#### Support vector machine

SVM [38] is used to build a classifier discriminating miRNA targets interactions with high quality. SVM is a supervised machine learning algorithm, aiming to solve linear and nonlinear classification and regression problems. It affords a mapping of the sample vectors into a non-linear, high-dimensional feature space, in which the samples may be separated by an optimal hyperplane. The similarity function between pairs of samples is called a kernel. In our study, a radial basis function (RBF) kernel is chosen for its higher reliability in finding optimal classification solutions over the other three kernels [39]. Let us denote 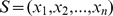 as a set of miRNA target data to be trained, each *x_i_* is an element of all possible miRNA target *X*. To form a SVM model, the data set *S* is represented as the set of features, 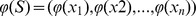, where 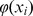 can be defined as a real-valued vector. Then SVM is designed to process a set of pairwise comparisons 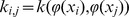, which is represented by an *n*×*n* matrix, used as input data of the RBF kernel:
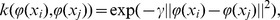
where the parameter 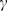 determines the similarity level of the features so that an optimal classifier can be constructed. The whole SVM approach is implemented with the Libsvm library [40].

#### Biologically relevant data set

Proposed classification system identifies real miRNA-target interactions from false positive candidates predicted by online predictors. Therefore, the positives of training dataset should be composed of experimentally verified *Arabidopsis thaliana* miRNA-target interactions. We retrieved 99 non-repetitive *Arabidopsis thaliana* positives from previous studies, which contain particular information of target site (Table S1). All of them work as positive training dataset.

To gain negative training dataset (including feature similarity with real miRNA-target interactions but tends to be false positives), a method proposed in previous study [34] was employed. miRNA targets predicted by the single method, which are not supported by other predictors, are frequently less credible than those identified by multiple methods. This paradigm was also analyzed in detail and proved in our results. Thus, miRNA targets supported by no more than one predictor were collected and 99 of them were randomly selected as negative training dataset. The ratio of positives to negatives is set to 1∶1 in order to maintain the balance of the classifier. We recognized that the selection method of negative training dataset may decrease the classifier accuracy slightly to some extent. This is because some positive elements may be included in the negative training dataset, while the performance of the classifier is commendable as discussed in the results.

#### SVM features

**Figure 2:**
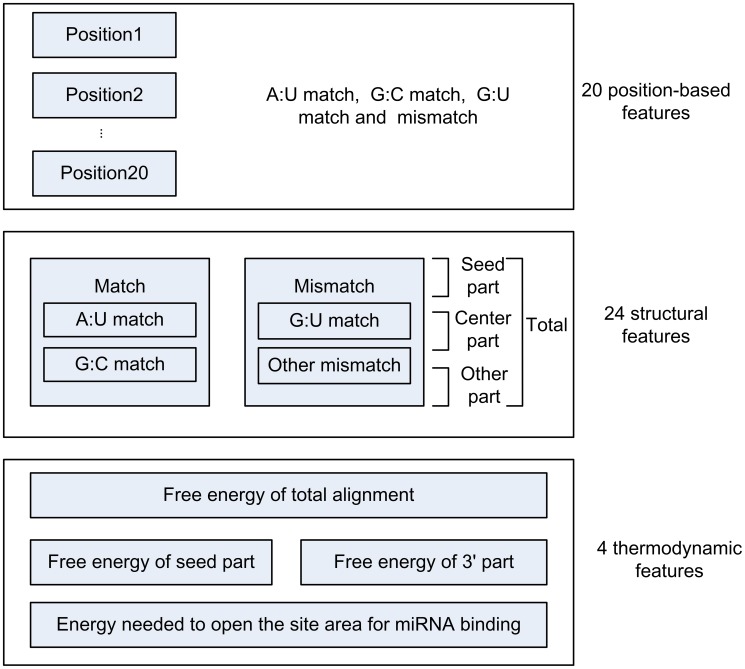
Three categories of SVM features. A total of 48 features belonging to 3 categories are extracted to classify high quality miRNA target interactions from false positive ones. All features mentioned are widely accepted to predict miRNA-target interactions and discriminate creditable targets from false positive ones.

It is a great challenge to extract a suitable feature set on which the classifier can be trained to identify both positives and negatives effectively. Features extracted from the proposed approach can be categorized into three classes: position-based features, structural features and thermodynamic features. The general features of 48 miRNA-target interaction is shown in Figure 2. All values were normalized to the interval (0, 1).

Position-based features are vital in the seed region in *Arabidopsis thaliana* [41]. Some special cases show that a single point mutation could affect miRNA target pairing and inhibit the miRNA's function, although these changes cause only a small variation in the interaction free energy. Besides the seed region, some other peculiar sites are reported to have influence on target recognition, e.g. position 16 and position 19 [14]. In order to figure out the complexity of recognition mechanism between miRNAs and targets, position-based features were extracted from positions 1 to 20. The rest were discarded if existed. Four types of cases were considered here including an A∶U match, a G∶C match, a G∶U match, and a mismatch, given a value from 1 to 4.Structural features are another significant part in miRNA-target interactions [20]. In our research, the miRNA target alignment was divided into four parts including seed part, central part, other part and total alignment. Moreover, the number of the four basic match types mentioned above was counted in each part. Among them, the central part is the main difference between animal and plant miRNA target. In plants, central matches usually lead to the cleavage of the target gene and exclude translational repression. Central mismatches lead to translational repression because they prevent slicing [42]. However, this factor is not considered in animals. Besides, the number of total matches and mismatches from all the four regions was calculated. In this case, 24 features were obtained.

Some of the thermodynamic features used in our approach were calculated by the RNAfold program from the Vienna RNA Package [43]. A linker sequence “GGGAAALLLLLLUUUCCC” was used to connect subsequences from miRNA and mRNA to calculate the free energy in 5′ part, 3′ part and total miRNA: mRNA alignment structure. In the linker sequence, “L” does not match with any nucleotide and is used to prevent miRNA and mRNA nucleotides from sequence-specific linker sequences [44]. Furthermore, the other characters are designed to prevent unexpected alignment of short matches. In addition, the target site accessibility is proved to be another determinant for the prediction of miRNA targets [45], [46]. Our approach has also considered the secondary structure, calculated by the RNAup program in Vienna package, near the targets site. A larger sequence containing the target site, 70 nt upstream and downstream, totally 140 nt, from both sides was extracted. The reason for choosing 70 nt was that base-pairing interaction between nucleotides of secondary structure is unlikely to happen when it is separated by >70 nt [47]. We set the first nucleotide of transcripts as the start of the larger sequence if the first nucleotide of the target site is located closer than 70 nt from it. The same rule was used to obtain the end of the larger sequence under some special circumstance. Then the energy needed for miRNA binding to open the site area 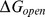 was calculated by RNAfold [48]. Thus, we gained 4 features.

#### Semi-Supervised Self-Training

Semi-supervised self-training is a method which trains the model with a small number labeled data and an additional set of unlabeled data. It reduces the effort needed to prepare the training set and maintains the stability of model in one sense. A previous study [49] has demonstrated that a model trained in self-training manner achieves results comparable to a model trained using a much larger set of fully labeled data. Our research meets this limitation as to study plants miRNA target interactions are not mature and we only got 198 samples in our training set.

As described in the previous study, miRNA target interactions from the IS set of prediction results have higher reliability to be real ones and miRNA target interactions from the OS set tend to be false positives. Firstly, weak labels +1, 0 and −1 are given to miRNA target candidates from the IS, MS and OS sets respectively. And results from them tend to be credible, suspicious and false positive. Samples with original label 0 are discarded during the set expansion step of self-training for the higher uncertainty in the training set. Then, the expansion rules of training set are defined as the following:

1. If samples with original weak label +1 are predicted to be positive, they can be used to expand the positive training dataset.
2. If samples with original weak label −1 are predicted to be negative, they can be used to expand the negative training dataset.

By this way, we take advantage of priori knowledge of results from the predictors. Consequently, satisfied stability and reliability of the classifier can be achieved.

**Figure 3:**
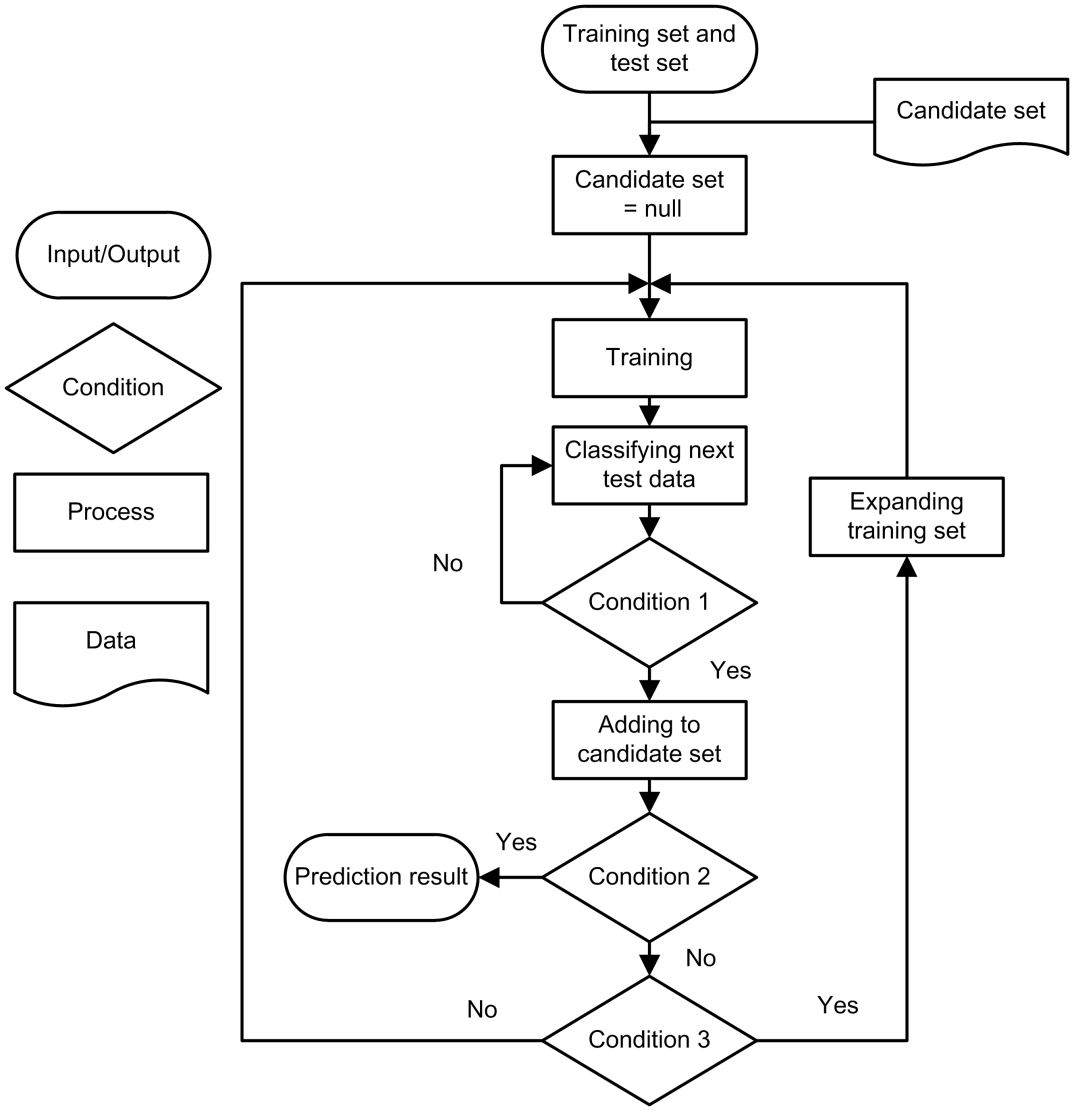
The process of semi-supervised self-training. The whole approach can be generalized into an iteration process of training and predicting. Condition 1 tells if the sample confirms to the expansion rules. Condition 2 tells if all unlabeled data in test set are labeled by the classifier. Condition 3 tells if the candidate set contains samples with positive label and negative label at the same time.

Figure 3 shows the process of semi-supervised self-training used in our approach. During the process, a candidate set is used to store samples needed to expand the training set and is initially set to empty. Firstly, a classifier model is trained in terms of the original training set, 198 samples in our research. Secondly, a sample from the test set is examined by the trained model and is assigned for a label 1 or −1. Further, the newly labeled sample is added to the candidate set if it satisfies the first condition, which tells if the sample confirms to the expansion rules mentioned above. Then we check the second condition. If all samples from the test set are labeled, the process enters the end state and output final results. Otherwise we go on to next condition. If the candidate set contains at least one positive sample and one negative sample, two samples with different labels are picked and added to the training set for expansion and removed from the candidates. This strategy ensures the balance of training set by adding samples with a ratio of 1∶1. No matter whether this condition is met, the process will move to the first step.

The whole method is an iterative for training the SVM model and expanding the training set. The model trained by the limited number of labeled samples will be more and more stable during the process and influence of small training set will be reduced simultaneously.

#### Feature subset selection

For pattern recognition, feature compression or extraction usually plays an important role. We employed principal component analysis (PCA) and constructed a PCA-SVM model to solve the problem caused by dependent or noisy features which lead to slower convergence and loss of accuracy of the classifier.

PCA is an unsupervised linear analysis method used for information extraction and dimension reduction [50]. It allows reducing the dimensionality of the problem through a linear transformation and producing a new set of variables/features, which is called “principal components” (PCs). PCs constitute a set of linear combinations of variables which preserves maximal amount of information with minimal redundancy. Here, “maximal amount of information” means the best lease-squares fit, or maximal ability to expound the variance of the original data. It can be expressed as below:
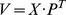
where 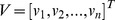 is the translated PCs; 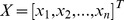 represents the set of original variables and *P* is the covariance matrix.

Furthermore, the column vectors (*P_i_*) of the coefficient matrix *P* are the eigenvectors of the covariance matrix (*S*), which is gained after normalization 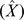.

To obtain the data matrix 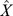 for a data set which has *N* observations and *n* variables, the observed sample matrix *Z* is normalized as bellow:
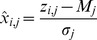
where *z_i,j_* represents an element of *Z*; *M_j_* denotes the mean value of *j*th variable and 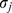 is the standard deviation of *j*th variable. Then the covariance matrix *S* is obtained as bellow:
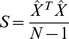
Usually, any column of *P* meets the following requirement, 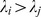 (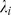 is the eigenvalue of the *i*th PC and *i*<*j*). When the cumulative percent variance of the first 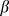 eigenvalues 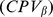 reaches or crosses a threshold and the usage of PCA reduced dataset reaches the optimal performance, the first 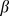 PCs are kept as the new feature space (i.e., the signal subspace):
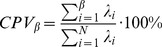



#### SVM Training

The performance of the SVM classifier was evaluated using 5-fold cross-validation performance. Accuracy is employed here as the evaluation criteria given below:
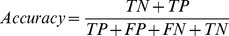
Where *TN* is the number of the predicted true negatives, *TP* is the number of the predicted true positives, *FP* is the number of the predicted false positives and *FN* is the number of the predicted false negatives.

The grid selection approach from the LIBSVM library was used to get the best parameters, *C* and γ. Then, a new SVM model was trained. Moreover, we sealed the whole data set in the interval (0, 1).

## Results

### Performance of online predictors

**Figure 4:**
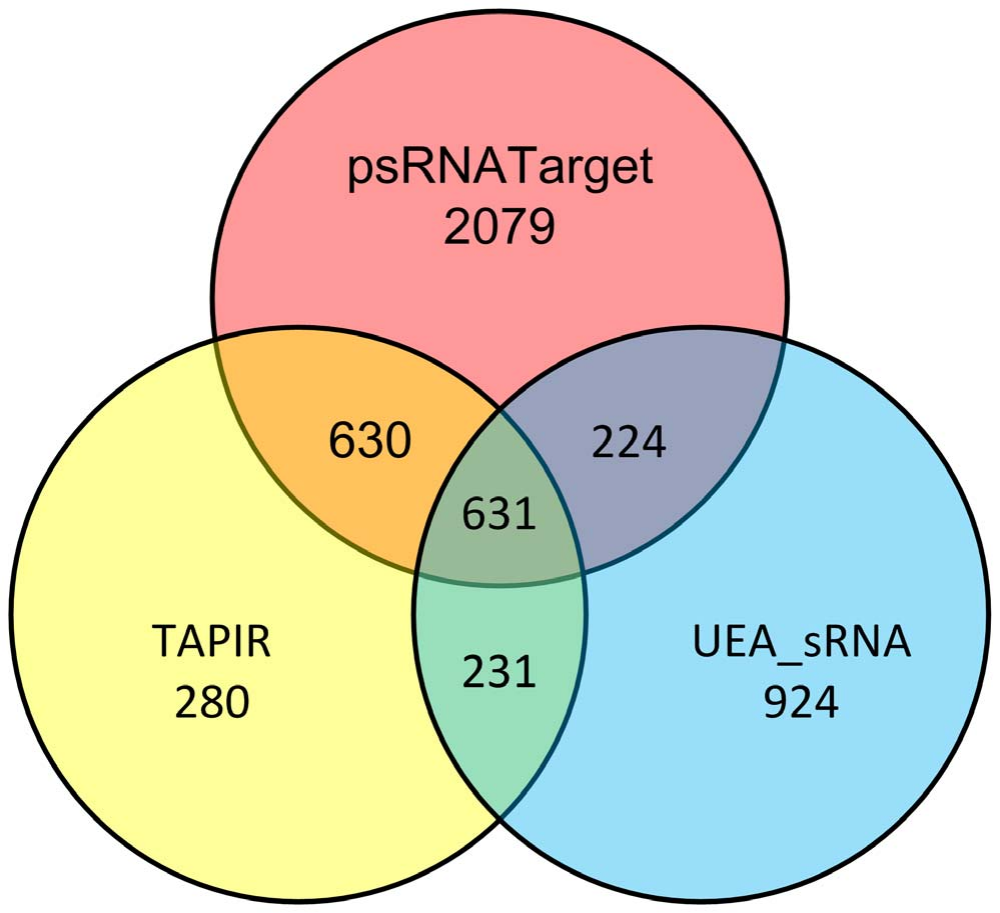
Statistical result of predictors in the proposed model. In order to analyze the performance of predictors chosen in our approach, we show the candidate set separately including overlaps between two predictors or among three predictors.

The statistical result of predictors used in the proposed model running on 338 *Arabidopsis thaliana* mature miRNAs and TAIR9 are shown in Figure 4, and detailed information is given in Table S2. Among them, psRNATarget provides the largest set (3564 candidate targets) because of its relatively less attention to the seed region and looser rules used within it, while TAPIR predicts the least (1772 candidate targets).

99 experimentally validated miRNA-target interactions are employed as reference set to evaluate the performance of online predictor. Results are shown in Table 2. OS has a large candidate set (3283/4999) as the fact of the various factors focused by different methods, and IS identifies the least (631/4999). Inconsistency between different predictors has been widely acknowledged and it is obviously greater in our approach according to the statistics. We studied current tools and picked the ones with less similarity in order to cover more candidates when online predictors were used. Then the results can be readily accepted. Assuming that the reference set we used covers all the true miRNA-target pairs, the true positive percentage in WS is bigger than any of the three predictors as we expected.

**Table 2:** Performance of predictors.

Method or Subset	Total^a^	Pos.^b^	P (%)^c^	p-value^d^
**psRNATarget**	3564	63	63.6	4.31E-13
**TAPIR**	1772	63	63.6	1.068E-3
**UEA_sRNA**	2010	49	49.5	4.02E-07
**OS**	3283	7	7.1	1.53E-40
**MS**	1085	21	21.2	5.33E-07
**IS**	631	42	42.4	1.00E+00
**WS**	4999	70	70.7	5.13E-19

a Total number of predicted candidates in each subset.

b Number of experimentally validated miRNA-target interactions identified in each subset.

c True positive percentage.

d p-value calculated with IS.

Moreover, true positive percentages in OS, MS and IS turn out to be an ascending sort order, to the opposite, they decrease in the column of false positive percentage. At the same time, true positive gaps between each two subsets are extremely large (7.1% versus 21.2% and 21.2% versus 42.4%). It is obvious that miRNA-target interactions identified by multiple predictors are more credible than single predictor did. This shows the superior of our proposed method for the negative training dataset used for SVM model. The low percentage (7.1%) of true positive in OS makes it much unreliable for identifying miRNA targets. So, miRNA-target interactions identified by a single predictor can be approximately regarded as ones with negative features.

Among three predictors, TAPIR and psRNATarget identify more true targets (63/90); whereas UEA_sRNA identifies a little less (49/90), probably due to the stringent parameters and special hybridize energy ratio used in it. Anyway, these results demonstrate the reliability of traditional methods based on statistics. However, most targets are not experimentally validated. For one reason, the high false positive percentage is not avoided by traditional prediction methods of statistics. The other reason might be less of reference targets set used in this analysis. This is the primary reason why we introduced machine learning method to face the challenge of searching more qualified miRNA-target interactions in plants.

Moreover, a chi-square testing was conducted in light of IS. As can be seen from the *p-values*, all subsets have large difference to IS with *p-values* close to 0, which reflects significant difference with IS. Thus, we conclude that IS is much better than any other sets.

### PCA Feature Extraction

**Figure 5:**
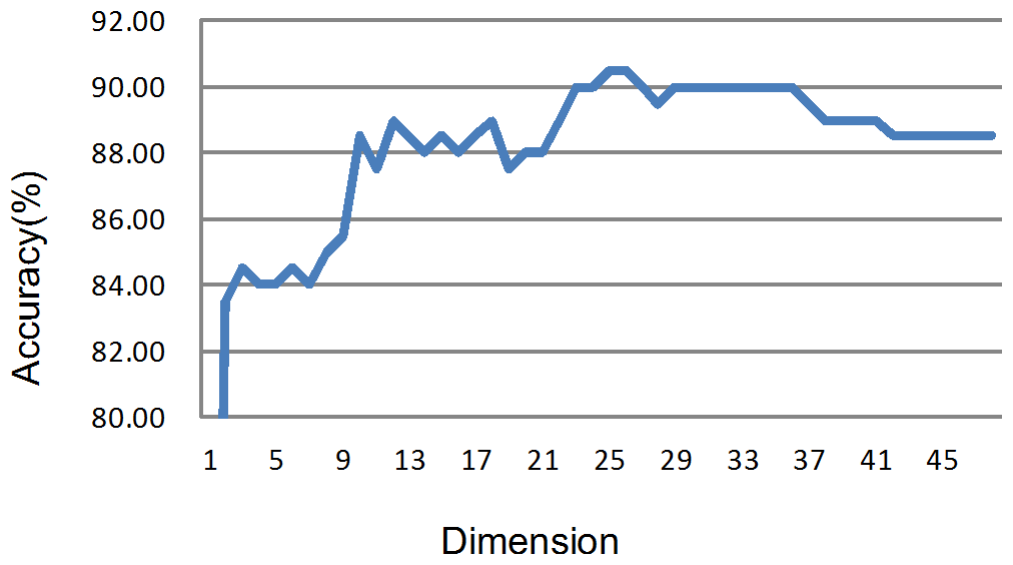
Diagnostic accuracy due to reduced dataset dimension using PCA. A 5-fold cross-validation approach is repeated for 48 times from 1PC to 48 PCs to view the change of accuracy and get the optimal dimension.

For the proposed miRNA-target interactions prediction system, 48 features, including 20 position-based, 24 structural and 4 thermodynamic features described in the “SVM features” section, were extracted. The whole training set with 99 positive samples and 99 negative samples were used for PCA for further feature extraction. Optimum diagnostic accuracy results due to PCA reduced dimension are given in Figure 5. The coefficient matrix *C* is shown in Table S3.

The optimal performance was researched using the first 25 PCs. Therefore, the original 48 features were reduced into 25 new ones which are uncorrelated. Next, the data with 25 PCs were used to train the SVM classifier model, replacing the original 48 features. The remaining 23 components were discarded, which contribute least to classifier.

### Kernel Selection

**Figure 6:**
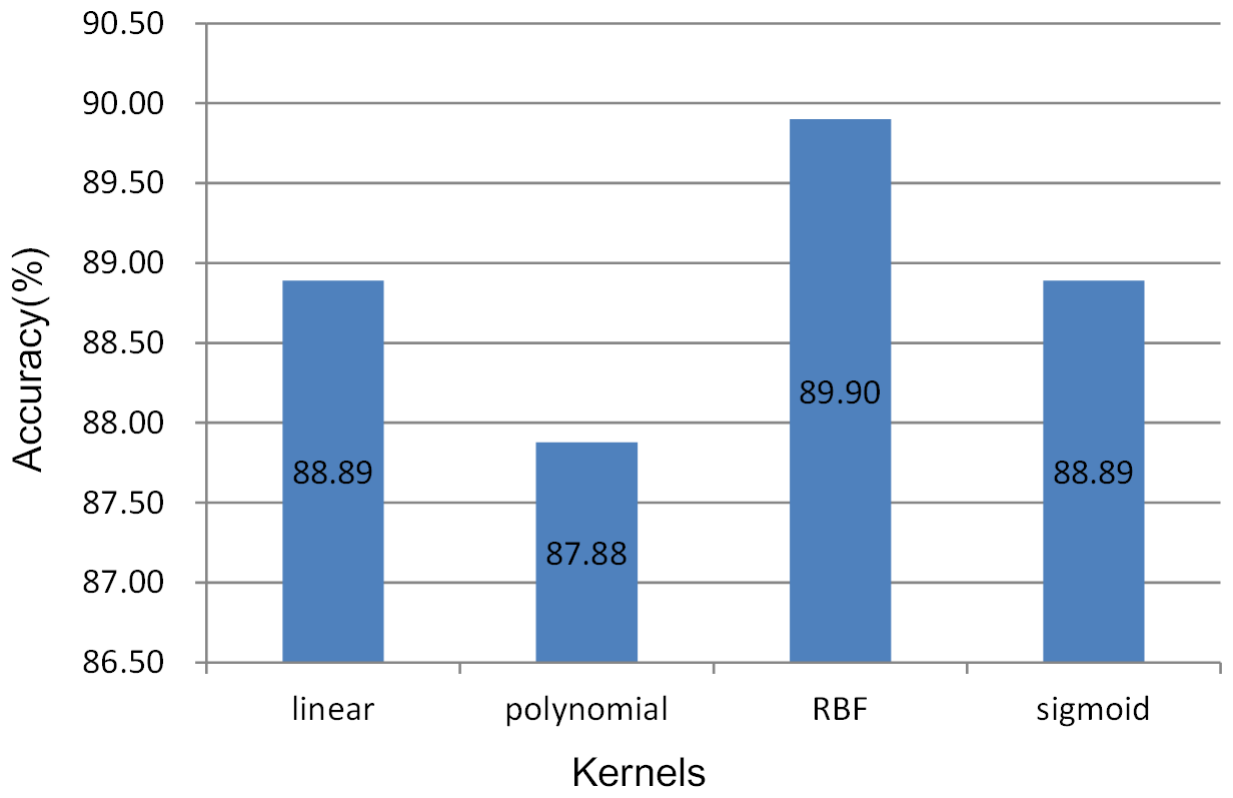
Leave-one-out cross-validation on four kernels. The cross-validation approaches for different kernels were run on our training set including 198 samples. The accuracy was used to evaluate the performance.

Using SVM, it is necessary to find the optimal kernel over a given set of kernels. A leave-one-out cross-validation approach was conducted on our training set using four different kernels including linear kernel (linear), polynomial kernel (polynomial), radial basis function kernel (RBF) and sigmoid kernel (sigmoid). A leave-one-out cross-validation involves using a single observation from the training set as the validation data, and the remaining observations as the training data. This is repeated so that each observation in the sample is used once as the validation data. Results are shown in Figure 6. RBF was selected for the accuracy of 89.9%, more than one percentage over the other three kernels.

### Negative Training Set Evaluation

A contrast test was conducted to further prove the rationality of our method to pick the negative training dataset. Four SVM models were built using different negative training datasets randomly selected from different sets of IS, MS, OS and WS of candidate miRNA targets. They were predicted by three predictors. Then, a leave-one-out cross-validation approach is conducted using four training datasets separately. Results are shown in Table 3. The cross-validation with negatives from OS gains the highest accurate of 89.9%. This means that negatives from OS tends to have false positive features and can be better classified by the SVM model. While the cross-validation with negatives from IS was the lowest 57.1%, indicating that negatives from IS have features similar to the real ones and can be hardly separated. Besides, the results of WS and MS respectively are 73.2% and 66.7%. This further supports the point that miRNA targets predicted by the single method are frequently less credible than those identified by multiple methods.

**Table 3:** Leave-one-out cross-validation using different negative training sets.

	IS	MS	OS	WS
**Accuracy(%)**	57.1	66.7	89.9	73.2

### Classifier performance

**Figure 7:**
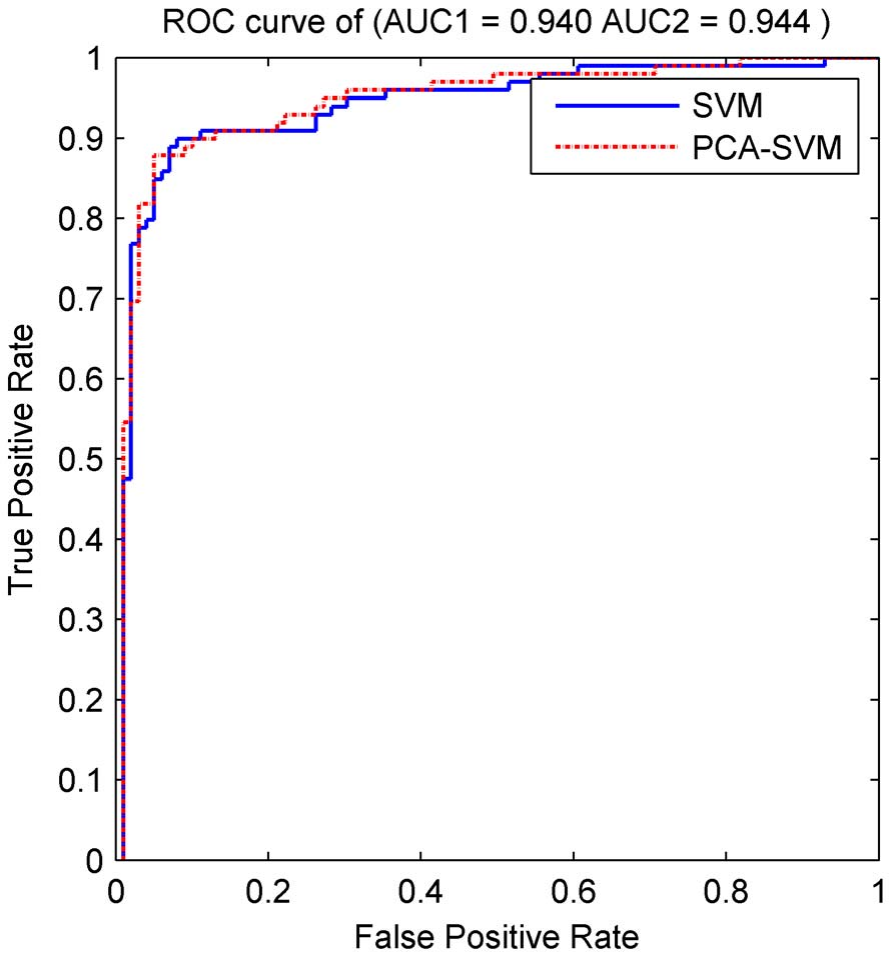
ROC curves of SVM and PCA-SVM. The ROC curves of classifiers created on 48 original features (the blue solid line) and 25 features after PCA (the red dotted line).

SVM classifier is implemented to filter the false positive miRNA target candidates, so that more credible information is kept. Samples processed by PCA were classified by SVM. To conduct a performance evaluation, 5-fold cross-validation method was performed. Firstly, SVM model was trained using four-fifth of the complete dataset. And the remaining one-fifth of the dataset was used to evaluate its performance. Then different combinations of training and testing datasets were repeated five times and the average of these five results was recorded as final result. We also repeated this process using samples with 48 original features to compare PCA-SVM with SVM model. Meanwhile, a simulative semi-supervised 5-fold cross-validation approach was conducted to show the contribution of semi-supervised self-training method. All candidates with weakly labels from the candidate set were randomly picked to expand the training set in each iteration process of 5-fold cross-validation. ROC curve, generated by the average *FP* and *TP* through Libsvm package, is hired to determine the cutoff value and performance of our classification model. Results are shown in Figure 7.

The area under the ROC curve of PCA-SVM model is 94.40%.It is almost the same as that of SVM (94.00%). Both of these two models have satisfied prediction capability. Whereas, the accuracy rate of PCA-SVM model has an increase of 2 percentage points over the SVM model shown in Table 4. Moreover, the accuracy of self-training model also increased by 2.5 percentage points over the SVM model. This indicates the positive influence of semi-supervised method to our classifier.

**Table 4:** Detailed information of SVM model and PCA-SVM model.

Method	Dimension	AUC (%)	Classification Accuracy (%)
**SVM**	48	94.00	88.50
**PCA-SVM**	24	94.40	90.50
**Self-Training**	48		91.00

### Parameter optimization

Before the PCA-SVM model was trained to classify all test sets containing both credible miRNA-target interactions and false positive ones, a grid search approach was conducted to obtain optimal parameters *C* and γ. Result shows that the accuracy of classification model reaches the maximum within the area *C* = 2^−1^ and γ = 2^−1^.

### Filter results

The prediction results for credible miRNA-target interactions are given in Table S4. We input all 4999 miRNA targets candidates into the classifier, 1942 of which were predicted to be positive. By analyzing the results set with prediction label 1. We found that 2573 out of 3283 candidates in OS are filtered with a ratio of 78.4%; 411 out of 1085 candidates in MS are filtered with a ratio of 37.9%; while only 73 out of 631 are filtered in IS with a ratio of 11.6%. All these consequences indirectly prove that miRNA targets predicted by single method are frequently less credible than those identified by multiple methods.

**Figure 8:**
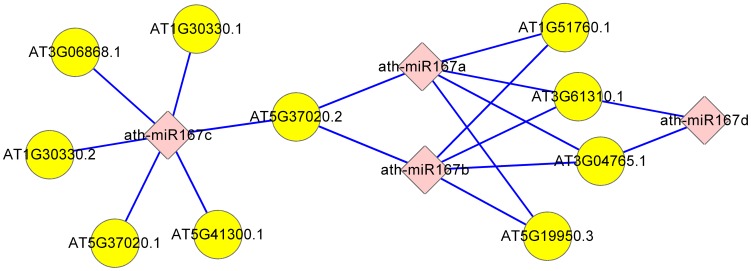
Partial interactive network of miRNA and targets. This partial network consists of 4 miRNAs from ath-miR167 family and 18 miRNA-target interactions. Diamond and circular nodes represent miRNAs and target genes respectively. An edge represents a targeted relation.

An interactive network was formed according to the outcome of PCA-SVM classifier in our approach. It describes credible interactions between *Arabidopsis thaliana* miRNAs and targets. A small portion of the network is shown in Figure 8. According to the results, 4 miRNAs from ath-miR167 family and 18 miRNA-target interactions were screened. Among them, the interaction with AT1G30330 and AT5G37020 are positive samples in our experiment. All these results are high quality interactions to ath-miR167 family gained by our integrated approach.

### Validation with degradome sequences

High-throughput sequencing-based methods have been widely used to detect RNAs containing miRNA-mediated cleavage of targets. This provides decent evidence for the prediction of miRNA-target interactions. Data from degradome sequencing cannot be used as training samples in our classifier. Because the specific binding sites of each miRNA are not strictly verified by experiments. However, they can serve as a large set of miRNA-target interactions to verify the prediction results for our approach. We first retrieved 1618 *Arabidopsis thaliana* miRNA-target interactions supported by degradome sequencing from starBase. Then two sets of miRNA-target interactions are predicted. One is obtained by using three online predictors respectively, and the other is obtained by using the combination of online predictors and local PCA-SVM classifier. We aim to match the two sets of miRNA target genes to those genes from degradome sequencing experiment.

We calculated the reliability values (*R-value*) of the candidate set, the final set as well as the true positive rate (*TP*) of the classification filter statistically to prove the good performance gained in our approach using the following formulas:
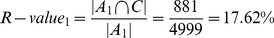


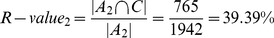


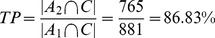
where *R-value*_1_ and *R-value*_2_ reflect the reliability of outcome sets predicted by online predictors and the whole approach, *TP* represents the true positive ratio of our PCA-SVM classifier; *A*_1_ and *A*_2_ denote the outcome sets from online predictors and the whole approach, in other words, *A*_1_ represents the aforementioned WS and *A*_2_ is a subset of *A*_1_ filtered by the PCA-SVM classifier; *C* denotes the set of 1618 *Arabidopsis thaliana* miRNA-target interactions data supported by degradome sequencing; 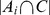 is the number of miRNA targets predicted which is supported by degradome sequencing data. In order to reduce the influence of one degradome data matching with multiple miRNA-target interactions, we ensure that each degradome data can only support zero or one miRNA-target interaction.

The *R-value* has an increasing number from 17.62% to 39.39%, almost 22 percentage points after PCA-SVM classifier was used. For *R-value*_2_, although our classification model is correct around 90%, it gets a value less than a half. On one hand, there is not an entirely accurate method in the miRNA-target interactions identification, even the degradome sequencing. On the other hand, computational prediction tools do produce false positive results which can be reduced, not completely removed, by our PCA-SVM model. Assuming that all 881 miRNA-target interactions represented by 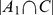 were real ones with clear function in *Arabidopsis thaliana*, our local filter approach only mistook 116 real targets, which represents by 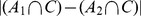, within the 3057 targets filtered, which represents by 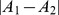. The error rate of 3.79% is low enough for the prediction approach. Meanwhile, *TP* proves satisfied performance of our classification filter from another perspective with a value of 86.83%. Moreover, throwing off this assuming, some of miRNA-target interactions from degradome sequencing experiment may be questionable. Thus, although we cannot accurately measure it, the sensitivity of our approach may be better than signed here.

miRNA-target interactions from the outcome of our approach supported by degradome sequencing data have proved the perfect performance, meanwhile, the other 1177 targets may be the advantage of our integration approach. The experimental results of our approach are worth of a deeper analysis and further biological study.

### Performance on other plant species

Many plant species have not been extensively studied so far. This means that the training data set of experimentally validated miRNA targets can be hardly found. Because of the similarity of miRNA target interactions between different plant species, we tend to filter miRNA-target interactions of other plant species using the proposed model constructed on *Arabidopsis thaliana* training set. The whole approach was carried out on *Oryza sativa* and *Vitis vinifera* to prove the usability of the proposed model. Additionally, similar validation with degradome sequences of *Oryza sativa* and *Vitis vinifera* is conducted. TargetFinder was adopted instead of UEA_sRNA for transcripts uploading permission. The contrast information is shown in Table 5 and detailed results are given as Table S5, Table S6.

**Table 5:** Contrast information between *Arabidopsis thaliana* and other plant species.

Species	miRNA^a^	Result_1_ b	Result_2_ c	R-value_1_ %	R-value_2_ %	TP %
***Arabidopsis thaliana***	338	4999	1942	17.6	39.4	86.8
***Oryza sativa***	708	9833	4087	10.4	20.9	83.9
***Vitis vinifera***	186	1372	651	18.2	33.9	88.4

a Total number of miRNA.

b miRNA target interactions gained by predictors.

c miRNA target interactions predicted to be positives by PCA-SVM model.

From the detailed information we conclude that the proposed approach also gains good effects over *Oryza sativa* and *Vitis vinifera* with the fact that more than half candidates filtered out and nearly doubled *R-value* according to the validation with degradome sequences. *TP* value over 85% proves good adaptability of the PCA-SVM model used in other plant species. Besides, our method will behave better if more experimentally proved training samples are given.

## Discussion and Conclusions

High-throughput sequencing technologies have developed rapidly and led to massive genetic data. Many miRNAs and miRNA targets have been identified under this circumstance. Computational prediction methods have made great attributions to this issue and machine learning algorithms are either developed or introduced to face this challenge. However, existing methods of miRNA targets prediction usually has inconsistent results and the reliability is not ideal enough. Therefore, three widely used tools and a PCA-SVM classifier with self-training strategy were integrated successfully to cover as many target candidates as possible and ensure the reliability of them at the same time. The validation experiment with degradome sequences showed that miRNA-target interactions predicted by proposed approach had huge increase in credibility, and thus worth to be further studied.

PCA-SVM machine learning method with self-training strategy was introduced in the prediction of plant miRNA-target interactions for the first time and 1942 credible miRNA-target interactions were identified for *Arabidopsis thaliana*. However, machine learning methods used in the prediction of plant miRNA targets are still immature as expected. Further work is still needed to find more compatible methods to solve the problem of lacking training samples.

## Supporting Information

Figure S1 **Detailed values of the parameters used in online predictors.**
(DOCX)

Table S1 **99** ***Arabidopsis thaliana*** **positives gathered from previous studies.**
(XLSX)

Table S2 ***Arabidopsis thaliana*** **miRNA target candidates predicted by psRNATarget,** TAPIR and UEA_sRNA.
(XLSX)

Table S3 **Coefficient matrix** ***C*** **used in our PCA analysis.**
(XLSX)

Table S4 **Credible** ***Arabidopsis thaliana*** **miRNA target interactions gained by our classification filter.**
(XLSX)

Table S5 **Credible miRNA target interactions of** ***Oryza sativa*** **.**
(XLSX)

Table S6 **Credible miRNA target interactions of** ***Vitis vinifera*** **.**
(XLSX)
