# Supplementary material for: Plant microRNA-Target Interaction Identification Model Based on the Integration of Prediction Tools and Support Vector Machine
Source: PLoS One. 2014 Jul 22;9(7):e103181. doi: 10.1371/journal.pone.0103181 (PMC4106887; doi:10.1371/journal.pone.0103181)
Supplement: Figure S1 — Detailed values of the parameters used in online predictors. (DOCX) [file pone.0103181.s001.docx]

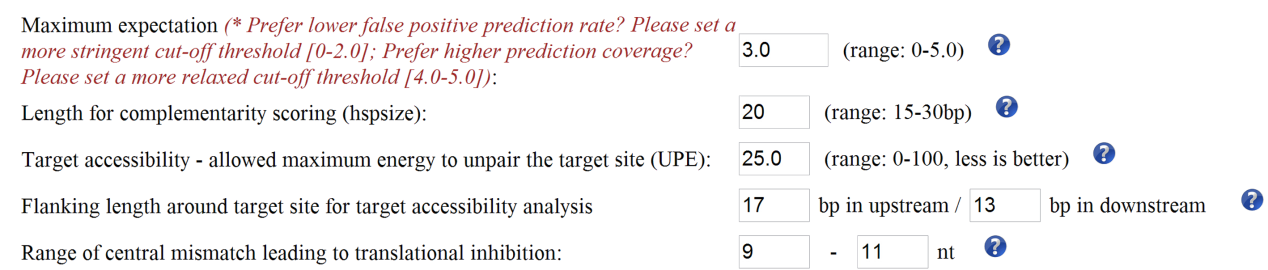


1. psRNATarget


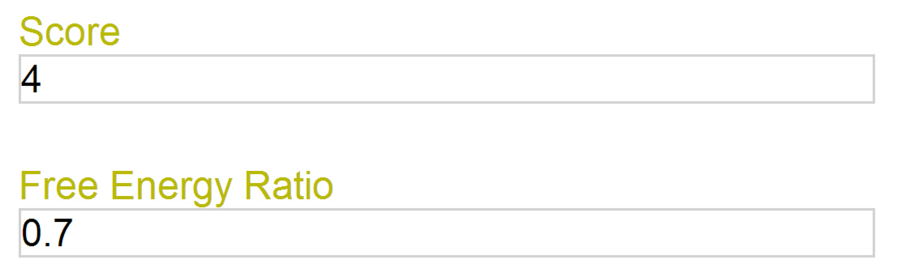


1. TAPIR

**Figure S1** Detailed values of the parameters used in online predictors psRNATarget

and TAPIR

.
